# LPCAT3 as a Potential Drug Target for Ultraviolet Radiation–Induced Cataract: Insights From Multiomics Analysis

**DOI:** 10.1002/kjm2.70219

**Published:** 2026-04-24

**Authors:** Fei Xu, Xiao‐Bo Wan, Yong‐Shun Liang, Tian Lan, Hao Liang

**Affiliations:** ^1^ Department of Ophthalmology The First Affiliated Hospital of Guangxi Medical University Nanning China; ^2^ Department of Ophthalmology Liuzhou People's Hospital Affiliated to Guangxi Medical University Liuzhou China

**Keywords:** ferroptosis, lens epithelial cells, lipid peroxidation, LPCAT3, ultraviolet B

## Abstract

Ultraviolet B (UVB) radiation is a major risk factor for cataract development, but the molecular mechanisms underlying this process, particularly the involvement of regulated cell death pathways such as ferroptosis, remain unclear. Transcriptomic, proteomic, and metabolomic analyses were performed on lens tissues from UVB‐induced cataract rat models and controls to identify differentially expressed genes (DEGs), proteins (DEPs), and metabolites. Integrated bioinformatic analysis was then used to identify key pathways and molecules. The role of the top candidate gene, lysophosphatidylcholine acyltransferase 3 (LPCAT3), was further investigated in vitro in rat lens epithelial cells (LECs) exposed to UVB and in vivo using an Ad5‐shLPCAT3 knockdown model. Multiomics analysis identified 1787 DEGs and 355 DEPs between cataractous and normal rat lenses. KEGG enrichment highlighted pathways including complement/coagulation cascades, cell cycle, mineral absorption, and glycerophospholipid metabolism. Metabolomic analysis revealed 2332 significantly altered metabolites enriched in sphingolipid, purine, and glycerophospholipid metabolism. Integrated analysis revealed that expression of LPCAT3, a key enzyme in glycerophospholipid metabolism, was significantly upregulated. UVB irradiation of LECs induced ferroptosis, which was characterized by decreased viability, increased ROS, MDA, 4HNE, and ACSL4 expression; decreased GPX4 expression; and upregulated LPCAT3 expression. LPCAT3 knockdown significantly protected LECs against UVB‐induced cell death, ROS production, lipid peroxidation, and ferroptosis marker dysregulation. Ad5‐shLPCAT3 injection attenuated UVB‐induced lens opacification, decreased ROS/MDA levels, and reversed ferroptosis‐related protein changes in vivo. This study demonstrated that LPCAT3 upregulation drives ferroptosis in LECs and that targeting LPCAT3 effectively mitigates UVB‐induced LEC ferroptosis and cataract formation in vivo, highlighting its therapeutic potential.

## 1 Introduction

Cataracts are the leading cause of blindness globally, making their pathogenesis and prevention a major challenge in ophthalmology [1]. Epidemiological studies have identified ultraviolet B (UVB) radiation as a primary environmental pathogenic factor that induces lens epithelial cell (LEC) damage and lens protein denaturation, ultimately causing visual impairment [2, 3]. Although surgical intervention remains the standard treatment [4], identifying early intervention targets to delay or reverse disease progression constitutes the core objective of fundamental research [5].

UVB irradiation stands out among cataract‐inducing factors because of its pervasive environmental presence and definitive oxidative damage potential [6]. UVB penetrates the cornea and aqueous humor, reaching the lens directly and elevating reactive oxygen species (ROS) levels in LECs [7]. Excessive ROS induces oxidative stress damage by attacking cellular macromolecules, including proteins, lipids, and DNA. This damage further dysregulates intracellular signaling pathways, induces apoptosis or necrosis, accelerates the degeneration and opacification of lens fiber cells, ultimately culminating in cataractogenesis [8, 9]. Ferroptosis, a novel form of regulated cell death, is increasingly recognized for its distinct oxidative lipid metabolism and iron dependence in multiple diseases. Its core feature involves iron‐dependent accumulation of lipid peroxidation damage, where elevated intracellular iron ions coincide with reduced glutathione peroxidase 4 (GPX4) activity, leading to an inability to clear lipid peroxides, progressive membrane damage, and eventual cell death [10]. Ferroptosis has been linked to cataracts [11]. As targeting ferroptosis may represent a novel therapeutic strategy for UVB‐induced cataracts [12], a more comprehensive understanding of the underlying mechanisms involved is imperative.

The pathogenesis of ocular diseases frequently involves multidimensional regulatory networks spanning genes, proteins, and metabolites. Conventional single‐omics approaches often fail to comprehensively capture dynamic interrelationships, propelling integrated multiomics to the forefront of complex disease analyses in contemporary science [13]. Integrated multiomics analysis overcomes gene‐protein expression disconnects caused by factors such as post‐transcriptional modifications and translation efficiency [14, 15]. Metabolomics further illuminates the functional impact of metabolites on gene expression and protein activity [14, 15]. This multitiered approach facilitates the reconstruction of disease‐specific molecular regulatory networks and provides critical insights into the mechanisms underlying pathogenesis. Multiomics approaches have elucidated the interactive network of glucose metabolism‐inflammation pathways in diabetic retinopathy [16], while integrated proteomics‐metabolomics analyses have revealed major alterations in inflammation‐related mediators and lipid metabolites in patients with glaucoma, suggesting their potential involvement in the disease [17]. To date, the mechanisms underlying UVB‐induced cataracts have not been analyzed using multiomics integration.

This study employed transcriptomic, proteomic, and metabolomic technologies to analyze ferroptosis‐related mechanisms in UVB‐induced cataract formation in the rat lens, with the aim of deciphering key regulatory factors and signaling pathways at the molecular level and providing innovative strategies for cataract prevention and treatment.

## 2 Materials and Methods

### 2.1 Animals

All animal experiments were performed in accordance with the Association for Research in Vision and Ophthalmology statement for the Use of Animals in Ophthalmic and Vision Research and were approved by our institution's Animal Ethics Committee. An equal number of male and female Wistar rats (2 months old, approximately 200 g) were obtained from SPF Biotechnology Co. Ltd. Prior to experimentation, the rats were acclimated for 1 week under standard conditions of 22°C ± 1°C, 45%–65% humidity, and a 12 h light/dark cycle, with ad libitum food and water.

### 2.2 Establishment of Rat Cataract Models

Wistar rats (*n* = 54) were randomly divided into two groups: the control group received standard feed without any treatment, and the UVB group was exposed to UVB irradiation for 7 days. For cataract induction, the rats were anesthetized, and their pupils were dilated using Mydrin‐P ophthalmic solution (Santen Pharmaceutical, Japan). Five min later, the rats received ocular exposure to a 308 nm UV lamp (1 × 10^3^ μW/cm^2^, equivalent to 10 W/m^2^) for 15 min daily across 7 days, achieving a cumulative dose of 9 kJ/m^2^. To prevent corneal infection, topical ofloxacin eye ointment (Shenyang Xingqi Pharmaceutical Co. Ltd., China) was administered, followed by a natural recovery period. The rats were euthanized, their eyes immediately removed, and their lenses dissected using an anterior approach.

### 2.3 RNA Sequencing (RNA‐Seq)

Total lens RNA was isolated using the RNeasy Fibrous Tissue Mini Kit (#74704; Qiagen, Valencia, CA, USA) following a standardized protocol. DNA was eliminated by incubation with RQ1 RNase‐free DNase (#M6101; Promega, Madison, WI, USA). RNA samples from both UVB‐exposed and control lenses were subjected to high‐throughput sequencing on an Illumina NovaSeq platform (San Diego, CA, USA). Each group comprised three biological replicates, with each replicate consisting of pooled lens capsules from six eyes to obtain sufficient biological material for multiomics analyses, given the limited tissue yield from individual lenses. For all samples, RNA‐seq reads were aligned to the reference genome using Hisat2 (≤ 2 mismatches allowed), followed by exclusive selection of uniquely mapped reads for gene expression quantification via the FPKM method. FPKM values were used to estimate normalized gene expression levels across samples. Differential expression analysis was conducted through the limma package, with statistically significant genes defined as those exhibiting log_2_|fold change| ≥ 1 and adjusted *p* value < 0.05.

### 2.4 Protein Extraction and Trypsin Digestion

For each group of lens samples, four volumes of lysis buffer were added, and the samples were lysed by sonication. After centrifugation (12,000 × *g*, 10 min, 4°C), the supernatant was transferred to a new tube, with subsequent protein quantification performed using a BCA assay kit (Beyotime, Shanghai, China). For proteomic analysis, three biological replicates per group were used, with each replicate consisting of pooled lens capsules from six eyes. Equal amounts of protein were digested from each sample. The mixture was brought to a consistent volume with lysis buffer supplemented with 5 mM dithiothreitol (Thermo Fisher Scientific, Waltham, MA, USA) and incubated at 56°C for 30 min for reduction. Subsequently, iodoacetamide (Selleck, Shanghai, China) was added to a final concentration of 11 mM, followed by incubation for 15 min at 25°C in the dark. To ensure that the urea concentration was < 2 M, the solution was supplemented with tetraethylammonium bromide (Bjbalb, Beijing, China). Trypsin was added at a 1:50 ratio (enzyme: protein, m/m) for overnight digestion. An additional aliquot of trypsin was then added at a 1:100 ratio (enzyme: protein, m/m), and digestion was continued for 4 h.

### 2.5 Liquid Chromatography–Mass Spectrometry Analysis

Following dissolution in solvent A (0.1% formic acid, 2% acetonitrile/water), tryptic peptides were subjected to separation on a home‐made reversed‐phase analytical column (25 cm × 100 μm i.d.). Separation was achieved on an EASY‐nLC 1200 UPLC system (Thermo Fisher Scientific) using a gradient: 0–22.5 min (6%–22% solvent B), 22.5–26.5 min (22%–34% B), 26.5–28.5 min (34%–80% B), and 28.5–30 min (80% B) at 700 nL/min constant flow, where solvent B contained 0.1% formic acid in 90% acetonitrile/water. Eluted peptides were ionized using a nanoelectrospray (2300 V) on an Orbitrap Exploris 480 instrument. Full mass spectrometry (MS) scans (350–1400 m/z) employed 60,000 resolution, whereas MS/MS employed high‐energy collisional dissociation fragmentation (27% normalized collision energy) with a 120 m/z start mass at 15,000 resolution. The automatic gain control target and maximum injection time were 1E6 and 22 ms, respectively. Data were processed using data‐independent acquisition‐next‐generation software (v.1.8). Spectra were searched against the *Rattus norvegicus* UniProt proteome (20,230,103, 47,945 entries), appended with reversed decoy sequences. Trypsin digestion, allowing ≤ 1 missed cleavage, was specified. Fixed modifications included N‐terminal methionine excision and cysteine carbamidomethylation. The peptide spectral match false discovery rate was set to < 1%.

### 2.6 Gene Ontology and Kyoto Encyclopedia of Genes and Genomes (KEGG) Functional Annotation

Gene ontology (GO) enrichment analysis covering biological processes (BP), molecular functions (MF), and cellular components (CCs), along with KEGG enrichment analysis of differentially expressed genes (DEGs) and differentially expressed proteins (DEPs), was conducted using ClusterProfiler (v4.6.2).

### 2.7 Metabolomic Analysis

For metabolomic analysis, three biological replicates were used per group, each consisting of pooled lens capsules from six eyes. Samples were weighed into 2 mL tubes, homogenized with 1000 μL of ice‐cold extraction solvent (75% [v/v] 9:1 methanol: chloroform, 25% H_2_O) using steel‐bead grinding followed by 30 min of sonication. After incubation on ice for 30 min and centrifugation, supernatants were collected, concentrated, and dried. Residues were reconstituted in 200 μL 50% acetonitrile containing 4 ppm 2‐chloro‐l‐phenylalanine (internal standard), filtered, and subjected to liquid chromatography‐MS (LC–MS) analysis.

LC–MS analysis was conducted using an EASY‐nLC 1200 UPLC system (Thermo Fisher Scientific) equipped with an ACQUITY UPLC HSS T3 column (150 × 2.1 mm, 1.8 μm; Waters) under the following conditions: column temperature, 40°C; flow rate, 0.25 mL/min; injection volume, 2 μL. The mobile phases for the electrospray ionization positive (ESI+) and negative (ESI−) modes were 0.1% formic acid acetonitrile (B2)/0.1% formic acid water (A2) and acetonitrile (B3)/5‐mM ammonium c (A3), respectively. MS detection was performed using an Orbitrap Exploris 480 mass spectrometer (Thermo Fisher Scientific) equipped with an ESI source. The instrument was operated with a sheath gas pressure of 30 arb, auxiliary gas flow of 10 arb, capillary temperature of 325°C, and spray voltages of 3.50 kV (ESI+) and −2.50 kV (ESI−). The MS1 scan range was set to 100–1000 m/z with a resolving power of 60,000 FWHM, while the MS/MS resolution was 15,000 FWHM. Data were acquired in both positive and negative ion modes. For metabolomic data analysis, metabolite identifier conversion was performed using the MetaboAnalyst web platform. Differential metabolites were subsequently screened with significance thresholds set at log_2_|fold change| ≥ 1 and *p* value < 0.05. KEGG enrichment analysis was conducted for these differential metabolites.

### 2.8 Multiomic Multivariate Analysis

For transcriptome‐proteome correlation analysis, we integrated both datasets to identify significant DEGs and DEPs. These were categorized based on expression trends and visualized using scatter plots with distinct colors for each category and reference line. Finally, GO and KEGG enrichment analyses were performed on genes and proteins demonstrating consistent expression patterns. Pathway‐level analysis and visualization of specific metabolic pathways were performed using the Pathview package (v1.38.0), integrating DEGs from the transcriptomic data.

### 2.9 Cell Culture and UVB Exposure

Rat primary LECs (#RAT‐iCell‐m004; iCell, Shanghai, China) were cultured in the specialized medium for rat primary LECs (#iCell‐m004‐002r; iCell) at 37°C with 5% CO_2_. The cells were then washed twice with prewarmed phosphate‐buffered saline (PBS). After removing the PBS, chilled PBS was added until complete submersion was achieved. The culture dishes were then transferred to ice and exposed to handheld UV irradiation (2 W/m^2^) for 10 min [18].

### 2.10 Lentivirus‐Mediated Transduction

Short hairpin (sh) RNAs targeting lysophosphatidylcholine acyltransferase 3 (*LPCAT3*; sh‐LPCAT3#1, sh‐LPCAT3#2, and sh‐LPCAT3#3) and a negative control shRNA (sh‐NC) were designed and synthesized by Tsingke (Jiangsu, China). Once the fragments were annealed and purified, they were integrated into a pLKO.1‐puro lentiviral vector (Sigma‐Aldrich). The shRNA vector, packaging plasmid psPAX2 (Addgene, Massachusetts, Cambridge, USA), and envelope plasmid pMD2.G (Addgene) were transfected into HEK293T cells using polyethylenimine (Thermo Fisher Scientific) to generate recombinant lentiviral particles. These particles were then utilized for cell transduction with polybrene (8 μg/mL; Yeasen, Shanghai, China). After exposure to lentiviral particles, LECs were assessed for successful transduction after an appropriate incubation period.

### 2.11 Cell Counting Kit‐8 (CCK‐8) Assay

Cells were seeded at a density of 5 × 10^4^ cells/well in triplicate. After 0, 12, 24, 36, or 48 h, the cells were incubated for 2 h with the CCK‐8 reagent (Boster, Wuhan, China) at 37°C. Optical absorbance was measured at 450 nm using a microplate reader (Thermo Fisher Scientific). The cell survival rate was calculated as follows: cell survival (%) = (OD_sample_ − OD_blank_)/(OD_control_ − OD _blank_) × 100.

### 2.12 Cell Apoptosis Analysis

Once incubation was complete, LECs were harvested, resuspended in binding buffer, and stained for 15 min in the dark at 25°C with FITC‐Annexin V/propidium iodide using an Annexin V‐FITC kit (Beyotime). Apoptosis was assessed using a FACScan flow cytometer (BD Biosciences) and analyzed using CytExpert 2.3 (Beckman, USA).

### 2.13 Validation of LPCAT3 Function in Rat Cataract Models

E1‐deleted recombinant adenoviruses were constructed using the AdEasy Adenoviral Vector System (Stratagene, San Diego, CA, USA) to generate pAd‐shLPCAT3 and pAd‐scramble vectors. Transfection of PacI‐linearized plasmids into HEK293 cells was performed using Lipofectamine 2000 (Thermo Fisher Scientific) according to standardized protocols. The resulting Ad5‐shLPCAT3 and Ad5‐scramble viruses were purified and concentrated using double cesium chloride gradient ultracentrifugation.

Thirty‐six rats were assigned to four subgroups: control, UVB, UVB + Ad5‐scramble, and UVB + Ad5‐shLPCAT3 (*n* = 9). UVB irradiation was applied to induce cataracts in the relevant groups. For LPCAT3 intervention, intravitreal injections of Ad5‐shLPCAT3 (10^8^ PFU/mL; 2 μL/eye) were delivered into the vitreous cavity 14 days prior to UVB exposure, with Ad5‐scramble virus serving as a negative control. Cataract severity was graded as follows: 0, clear lens; I, clear lens with scattered vacuoles in the cortex; II, slightly increased nuclear density, early capsular opacification, and increased cortical vacuoles; III, elevated nuclear density with patchy white opacification in the cortex; IV, complete opacification presenting as a uniform white lens [19]. Given the minimal protein yield from a single rat lens capsule, samples were prepared by pooling tissues from six eyes, providing three biological replicates per group for analysis.

### 2.14 Lens Morphologic Examination

Morphological changes involving the lenses were monitored using a slit‐lamp microscope (66 Vision Co., Suzhou, China) after UVB exposure. However, UVB‐induced corneal cloudiness hinders slit lamp examination of the lens, necessitating lens enucleation. These were examined and photographed under a stereoscope (Olympus, Tokyo, Japan).

### 2.15 Detection of ROS Levels

Intracellular ROS levels were assessed by dihydroethidium (DHE) staining (Beyotime). After incubation, LECs were washed with PBS and subsequently incubated with a 5 μM DHE working solution for 30 min at 37°C in the dark. Excess stain was removed by washing with PBS prior to fluorescence microscopy (Olympus).

Lens samples were homogenized in lysis buffer (Beyotime) containing phenylmethylsulfonyl fluoride (PMSF; Beyotime), centrifuged (14,000 *g*, 10 min, 4°C), and incubated with 5 μM DCFH‐DA probe (Invitrogen, D399; 1 h, 37°C). Relative fluorescence units (RFU) were quantified at 485/527 nm using a microplate reader and normalized to protein concentrations determined using the Bradford assay (Beyotime).

### 2.16 Detection of Malondialdehyde (MDA) Levels

The lenses and LEC lysates were centrifuged (12,000 *g* for 10 min) for protein quantification. MDA levels were assessed using a commercial kit (Beyotime) by mixing the samples with the working solution, followed by 100°C heating for 15 min. After cooling and centrifugation (1000 *g*, 10 min), supernatants (200 μL) were analyzed at 532 nm via a microplate reader for MDA level calculation.

### 2.17 Reverse Transcription‐Quantitative Polymerase Chain Reaction Analysis

The TRIeasyTM total RNA extraction reagent (Yeasen) was used to isolate total RNA from the lenses and LECs. RNA sample concentrations were evaluated using a Nano 2000 UV spectrophotometer (Thermo Fisher Scientific), and then RNA samples were reverse transcribed into cDNA using the Hifair advanceFast first strand cDNA synthesis kit (Yeasen). Next, we performed reverse transcription quantitative polymerase chain reaction (RT‐qPCR) using Hieff qPCR SYBR green master mix (Yeasen). PCR primers used were as follows: LPCAT3 F 5′‐GAGCCTTAACAAGTTGGCGAC‐3′; R 5′‐CCCCACCATACCATGCTACC‐3′ and β‐actin F 5′‐GGTGACCCCTCCCCTCTATT‐3′; R 5′‐CCTCCAGCATTGGTCACCTT‐3′. All samples were analyzed using the 2^−ΔΔCT^ method. Data were normalized to β‐actin expression.

### 2.18 Western Blotting

To extract total protein from the lenses and LECs, RIPA lysis buffer (Beyotime) supplemented with PMSF was utilized, and a bicinchoninic acid protein concentration detection kit (Beyotime) was used to quantify protein abundance. The proteins were then subjected to separation by 10%–12% SDS‐polyacrylamide gel electrophoresis and transferred to polyvinylidene fluoride membrane. After blocking the membranes in 5% skimmed milk for 2 h, a two‐step incubation process was conducted: primary antibodies were applied at 4°C overnight, followed by secondary antibodies at 37°C for 40 min. All antibodies prepared in antibody dilution buffer (Beyotime) are listed in Table 1. Protein bands were detected using an ultrasensitive ECL chemiluminescence detection kit (Thermo Fisher Scientific), and quantification was performed using ImageJ software.

**TABLE 1:** Antibodies used in western blotting.

Antibody	Manufacturer	Cat. No.
4HNE	Abcam	ab46545
β‐Actin	Abcam	ab8227
GPX4	Abcam	ab125066
ACSL4	Abcam	ab155282
LPCAT3	Affinity	DF12033
Goat Anti‐Rabbit IgG H&L (HRP)	Abcam	ab6721

### 2.19 Statistical Analysis

Data were graphed as means ± standard error of the mean with at least three biological replicates and analyzed using GraphPad Prism 9.0 software (GraphPad, San Diego, CA, USA). Data normality was assessed using the Shapiro–Wilk test. Differences among multiple groups were analyzed using one‐way analysis of variance, followed by Tukey's multiple‐comparison test. Statistical significance was set at *p* values < 0.05.

## 3 Results

### 3.1 GO And KEGG Enrichment Analysis of Transcriptomic Profiles in UVB‐Induced Cataract Rat Lenses

**FIGURE 1:**
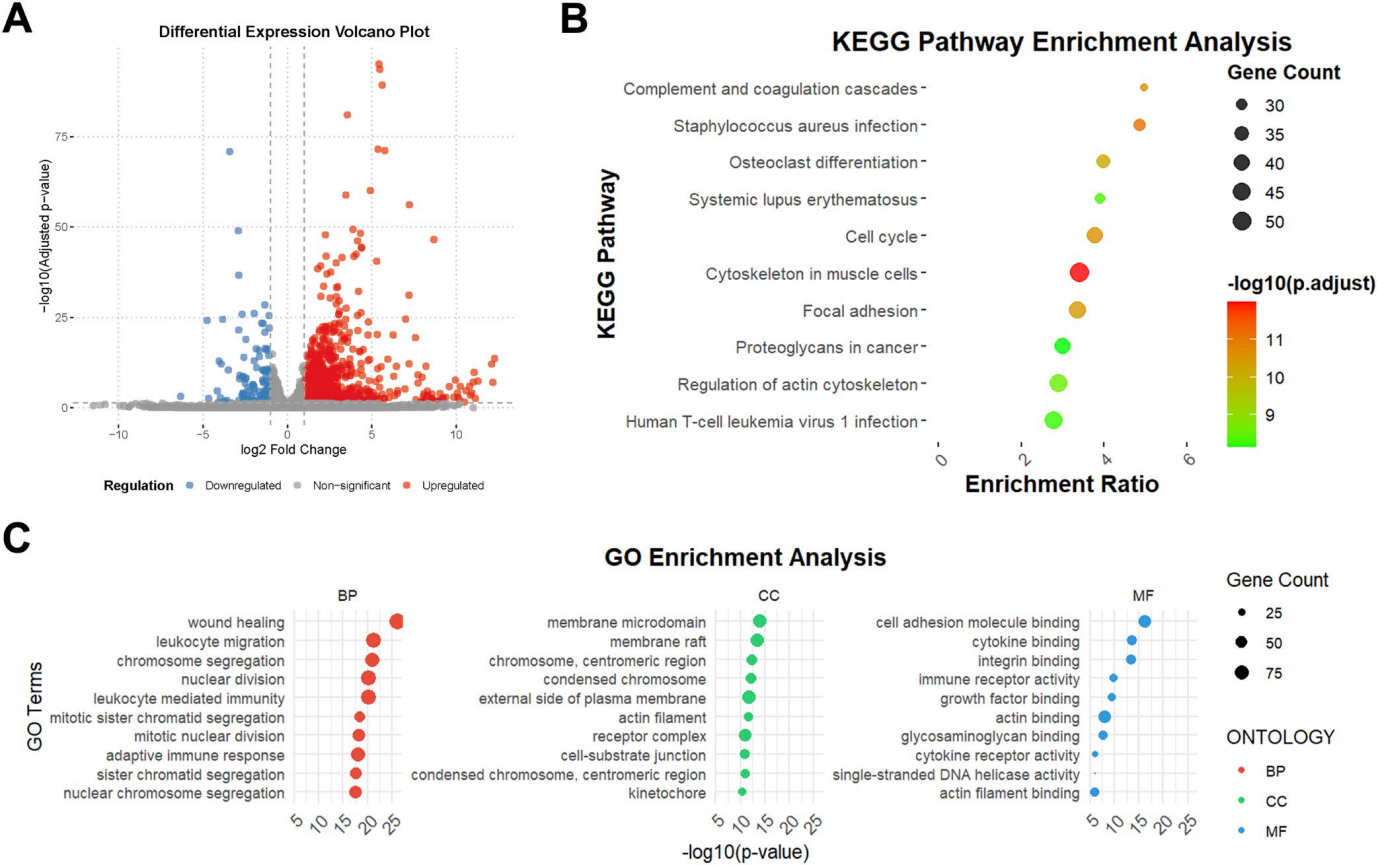
Transcriptomic Enrichment Analysis (GO/KEGG) in UVB‐induced cataract rat lenses. (A) Volcano plot displaying DEGs in cataract lenses versus normal lenses in transcriptomic analysis. (B) KEGG pathway enrichment of DEGs comparing cataract lenses with normal lenses using bubble plots. (C) Bubble plots displaying GO functional annotation analysis of DEGs across BF, CC, and MF.

To identify the DEGs between cataractous and normal lenses, transcriptome analysis was performed on lens tissues from normal and UVB‐induced cataractous rats. A total of 1787 DEGs were identified, comprising 1426 upregulated genes and 361 downregulated genes (|log_2_ (fold change)| ≥ 1, adjusted *p* < 0.05) (Figure 1A). KEGG pathway enrichment analysis of the DEGs revealed significant enrichment in pathways including cytoskeleton in muscle cells, *Staphylococcus aureus* infection, cell cycle, complement and coagulation cascades, and focal adhesion (Figure 1B). Functional annotation of DEGs using GO revealed significant enrichment across the three categories. In BP, DEGs were mainly associated with wound healing, leukocyte migration, chromosome segregation, nuclear division, and leukocyte‐mediated immunity. At the CC level, they were localized predominantly to the membrane microdomain, membrane raft, chromosome, centromeric region, condensed chromosome, and external side of the plasma membrane. For MF, key activities included cell adhesion molecule binding, cytokine binding, integrin binding, immune receptor activity, and growth factor binding (Figure 1C).

### 3.2 GO And KEGG Enrichment Analysis of Proteomic Profiles in UVB‐Induced Cataract Rat Lenses

**FIGURE 2:**
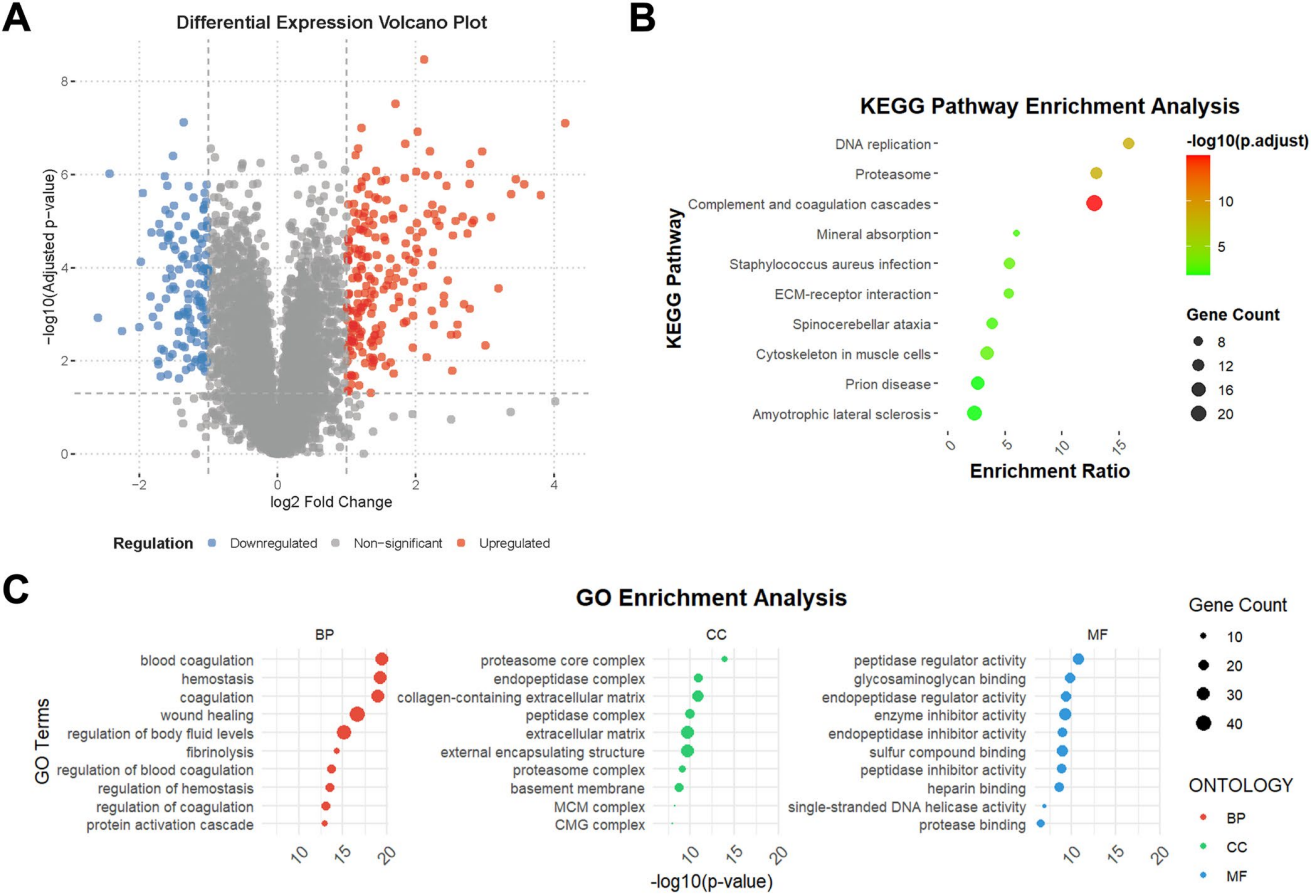
GO and KEGG proteomic enrichment in UVB‐induced cataractous rat lenses. (A) DEPs of cataract lenses versus normal lenses in proteomic analysis are displayed as a volcano plot. (B) KEGG pathway enrichment of DEPs comparing cataract lenses with normal lenses. (C) GO enrichment analysis of DEGs across BF, CC, and MF.

Proteomic analysis of lens tissues from normal and UVB‐induced cataract model rats revealed 355 DEPs. Among these, 216 were upregulated, and 139 were downregulated (|log2(fold change)| ≥ 1, adjusted *p* < 0.05) (Figure 2A). KEGG pathway analysis of the DEPs demonstrated significant enrichment involving the complement and coagulation cascades, DNA replication, proteasome, *S. aureus* infection, ECM‐receptor interaction, and mineral absorption pathways (Figure 2B). Significant enrichment across all three GO categories was observed for functionally annotated DEPs. DEPs were primarily linked to blood coagulation, hemostasis, coagulation, wound healing, and regulation of body fluid levels in BP. Predominant localization at the CC level was observed involving the proteasome core complex, endopeptidase complex, peptidase matrix, collagen‐containing ECM, ECM, and externally encapsulating structures. For MF, key activities were peptidase regulator activity, glycosaminoglycan binding, endopeptidase regulator activity, and enzyme inhibitor activity (Figure 2C).

### 3.3 Metabolic Profiling of Rat Lenses

**FIGURE 3:**
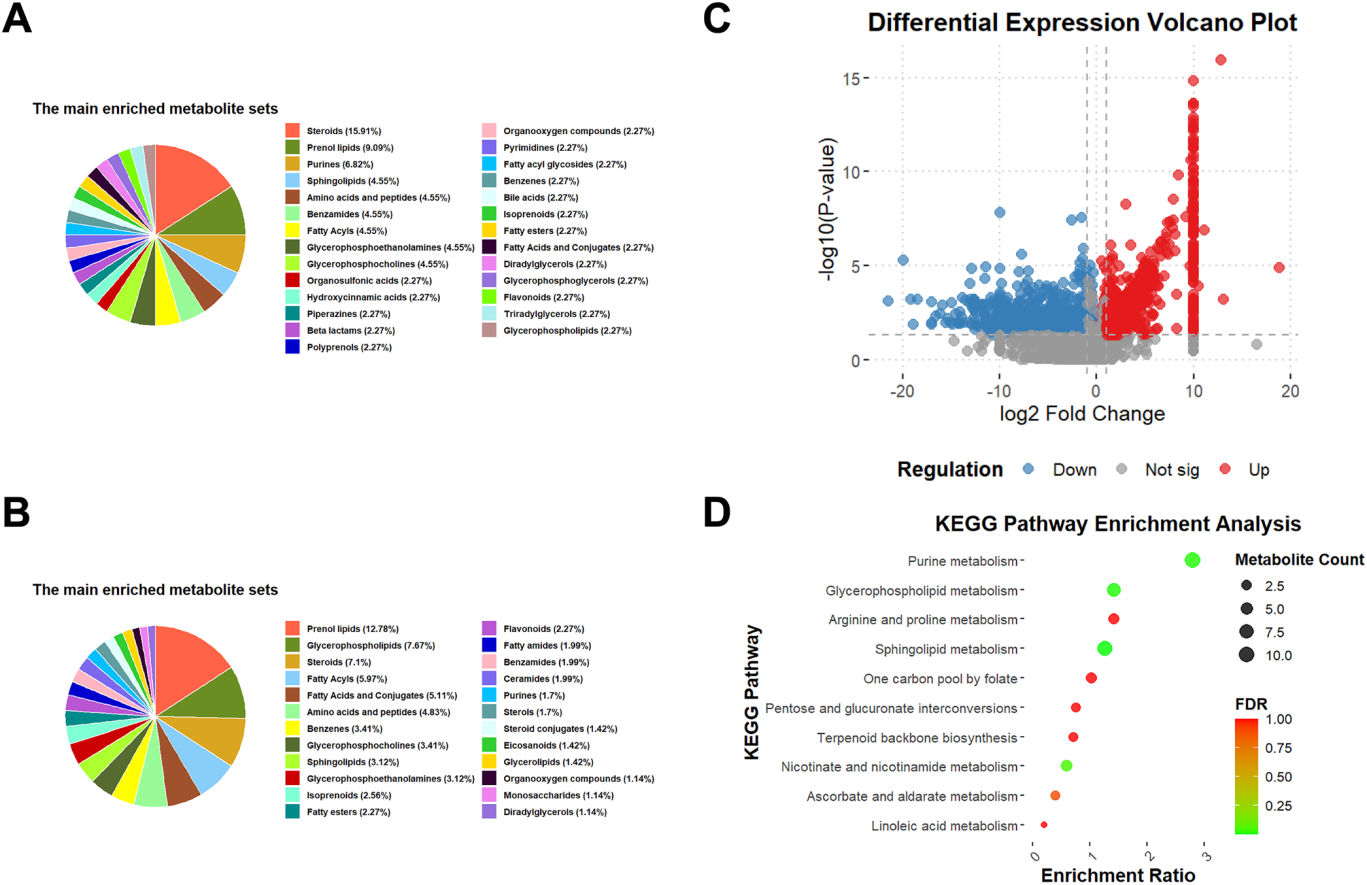
Metabolic analysis of UVB‐induced cataractous rat lenses. (A and B) Overall metabolite abundance distribution of cataract lenses in ESI+ and ESI− modes. (C) Volcano plot visualization of differential metabolites in cataract versus control lenses. (D) Bubble plot of KEGG pathway enrichment for differential metabolites.

Metabolomic analysis was performed by LC–MS. A total of 2332 metabolites exhibiting significant differences were screened in cataractous lenses compared to control lenses. Of the significantly altered metabolites, 1387 were downregulated, and 945 were upregulated. We further observed the overall metabolite abundance profiles in cataract lenses under both ESI+ and ESI− modes, as shown in Figure 3A,B. For cataract lenses in ESI+ mode, the metabolite classes with the highest relative abundances were steroids at 11.59%, prenol lipids at 9.09%, and purines at 6.82%. In ESI+ mode, metabolite classes with the highest proportions in cataractous lenses were prenol lipids (12.78%), glycerophospholipids (7.67%), steroids (7.1%), and fatty acids (5.97%). A volcano plot was used to depict the differential metabolites identified between cataract and control lenses, as presented in Figure 3C. Furthermore, metabolic pathway enrichment analysis revealed the predominant enrichment of differential metabolites in the following pathways: sphingolipid metabolism, purine metabolism, glycerophospholipid metabolism, and nicotinate and nicotinamide metabolism (Figure 3D).

### 3.4 Integrated‐Omics Analyses Reveal LPCAT3 as a Key Regulator of Ferroptosis in UVB‐Induced Cataracts

**FIGURE 4:**
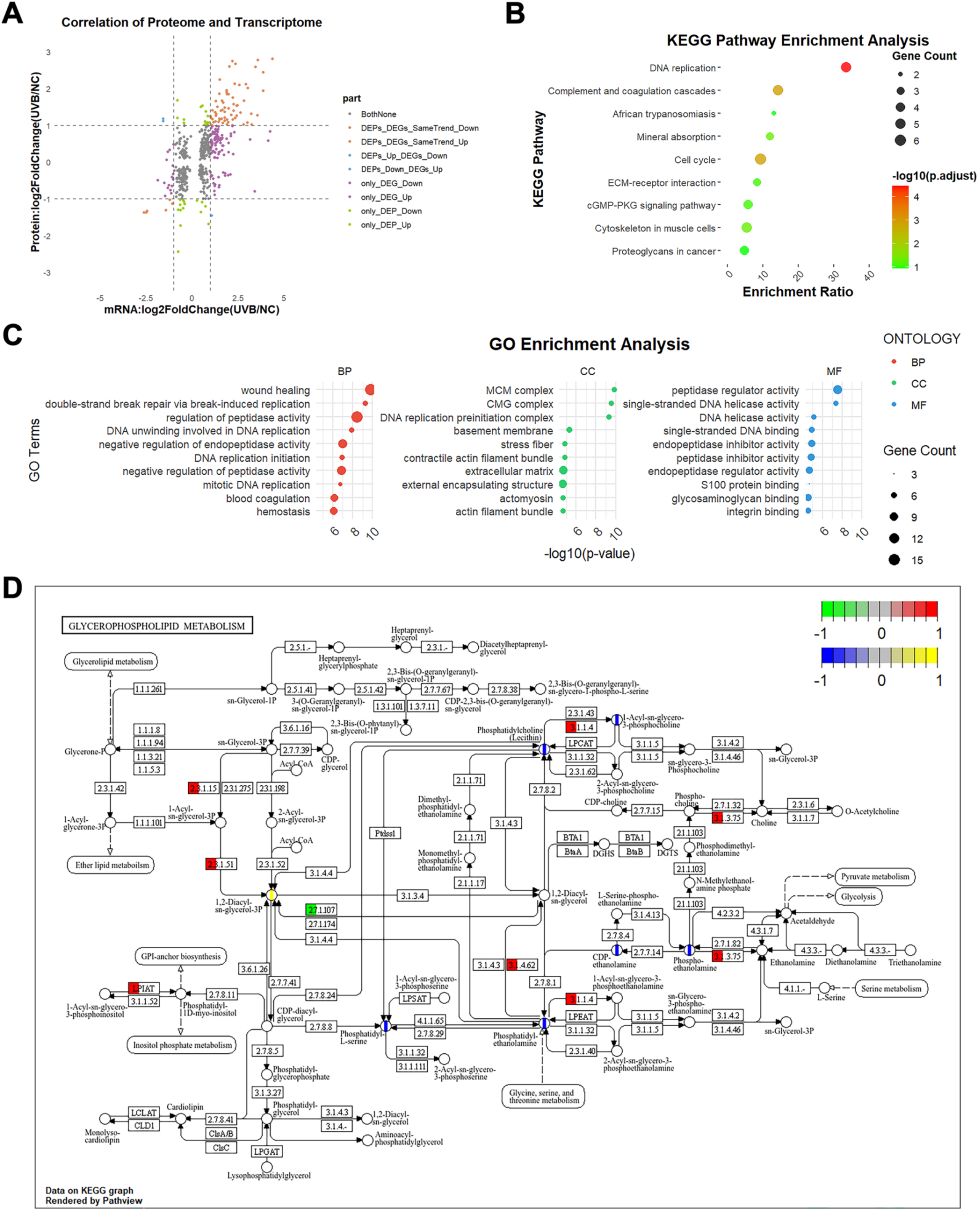
Integrated multiomics analyses of UVB‐induced cataracts. (A) Correlation analysis of DEGs and DEPs between control and cataract groups. (B) KEGG enrichment analysis of codifferentially expressed molecules in transcriptome and proteome data sets (C). GO enrichment analysis of codifferentially expressed molecules in transcriptome and proteome data sets. (D) Multiomics association analysis (metabolomics and transcriptomics) of cataract differential metabolites/genes related to glycerophospholipid metabolism.

To better explore the molecular mechanisms underlying cataract formation, we analyzed the DEGs and DEPs between control and cataract groups using correlation analysis. An overall workflow depiction of the integrated multiomics analysis is shown in Figure S1. Seventy‐four molecules were found to be differentially coexpressed in both the transcriptome and proteome, comprising six downregulated and 68 upregulated genes, indicating their regulation by a transcription‐translation cascade (Figure 4A). These molecules, which were differentially coexpressed in both the transcriptome and proteome, were predominantly enriched in the following pathways: DNA replication, complement and coagulation cascades, cell cycle, mineral absorption, and cytoskeleton in muscle cells (Figure 4B). Functional annotation analysis revealed that these molecules were primarily involved in the following processes: wound healing, double‐strand break repair via break‐induced replication, regulation of peptidase activity, DNA unwinding involved in DNA replication, and negative regulation of endopeptidase activity. At the CC level, these molecules were primarily localized to the MCM complex, CMG complex, DNA replication preinitiation complex, and basement membrane. MF was associated with peptidase regulatory activity, single‐stranded DNA helicase activity, single‐stranded DNA binding, and endopeptidase inhibitor activity (Figure 4C). Through integrated metabolomic and transcriptomic analyses, differential metabolites, DEGs, and associated metabolic pathways were identified in the control and cataract groups. Four coenriched metabolic pathways were identified: glycerophospholipid metabolism (Figure 4D), purine metabolism (Figure S2A), sphingolipid metabolism (Figure S2B), and nicotinate and nicotinamide metabolism (Figure S2C). Among these, the glycerophospholipid metabolism pathway has prominently captured our attention, as it is most directly implicated in ferroptosis [20]. As an enzyme responsible for esterifying unsaturated fatty acids to phosphatidylethanolamine in the glycerophospholipid metabolic pathway, LPCAT3 acts as a pivotal ferroptosis mediator [21]. Within the coenriched glycerophospholipid metabolism pathway, LPCAT3 (2.3.1.62) exhibited a significant increase in DEGs (Figure 4D). Given the important role of LPCAT3 in ferroptosis, we selected LPCAT3 for further investigation to examine its potential association with ferroptosis in UVB‐induced cataracts.

### 3.5 UVB Irradiation Induces Ferroptosis and LPCAT3 Upregulation in LECs

**FIGURE 5:**
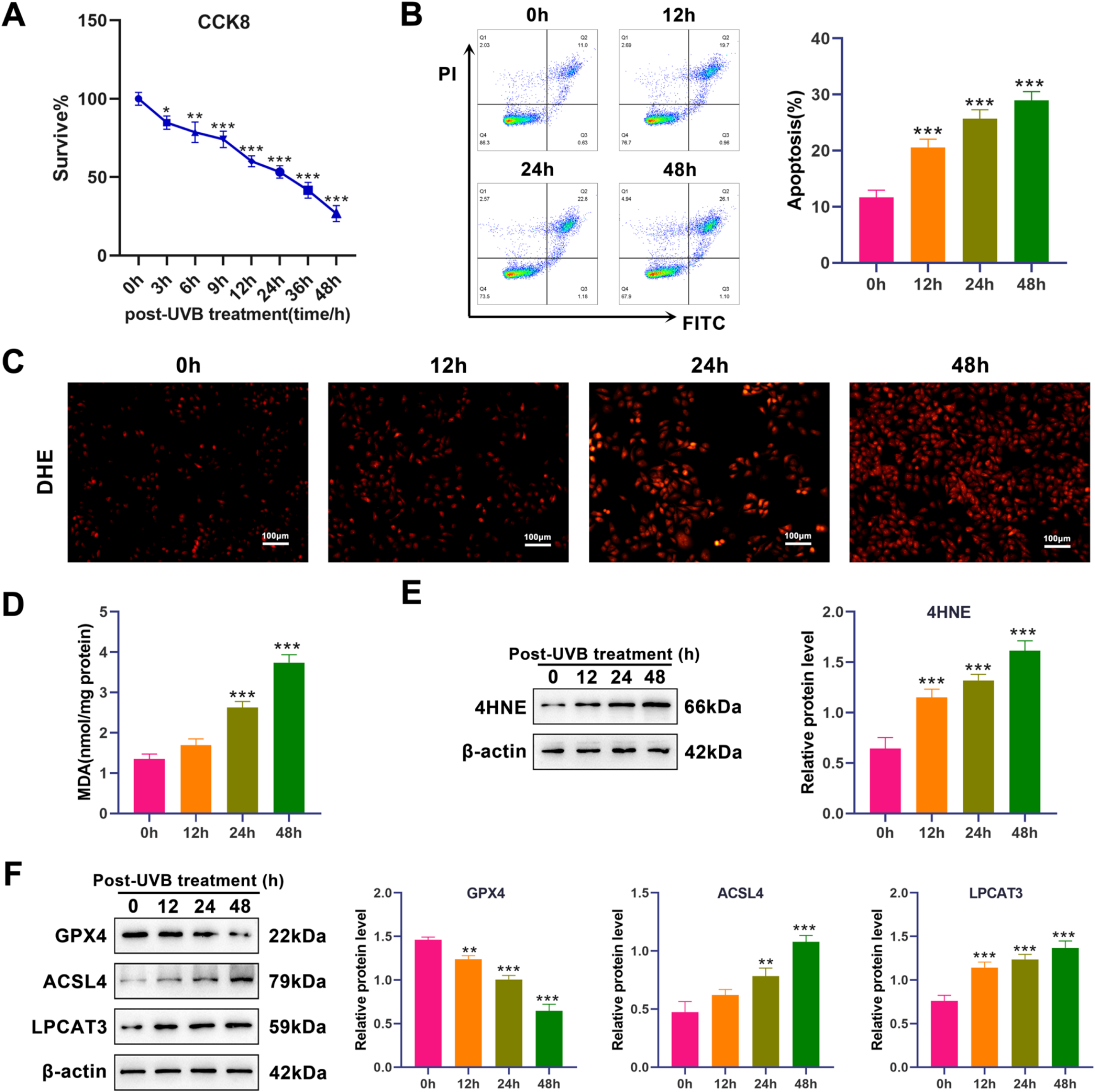
Ferroptosis induction and LPCAT3 upregulation are mediated by UVB irradiation in LECs. (A) After irradiation with UVB (2 W/m^2^, 10 min) and incubation for the indicated times, the survival rate of LECs was detected by CCK‐8 assays. (B–F) LECs irradiated with UVB, followed by incubation for 0, 12, 24, or 48 h. (B) Cell apoptosis assessed by flow cytometry assays. (C) ROS production analyzed after DHE staining. (D) MDA content measured using a commercial reagent kit. (E–F) Protein levels of 4HNE, GPX4, ACSL4, and LPCAT3 were detected by western blotting. One‐way ANOVA and Tukey's post hoc test were performed to determine statistical differences (A, B, D, E, F). Data are presented as the means ± SEM. *p < 0.05, **p < 0.01, and ***p < 0.001.

To investigate the effects of UVB irradiation on LECs, we measured cell survival rates using CCK‐8 assays at multiple time points after irradiation (2 W/m^2^, 10 min). A time‐dependent reduction in LEC survival rate was observed after UVB irradiation. After 48 h of UVB irradiation, the survival rate decreased to less than 40% (Figure 5A). To determine the time point at which cell survival declined significantly, we analyzed the apoptosis rate of LECs at 0, 12, 24, and 48 h post‐UVB exposure. As shown in Figure 5B, LEC apoptosis increased with incubation time, but the changes were not statistically significant from 24 to 48 h. Interestingly, cell viability continued to decrease significantly, but no significant change in apoptosis was observed during the 24–48 h period, indicating that other forms of cell death likely became predominant in the later phase. Given the role of LPCAT3 as a ferroptosis‐related protein, we further analyzed changes in ROS levels in LECs after UVB irradiation. DHE staining revealed a significant increase in ROS levels after prolonged incubation (Figure 5C). Furthermore, the key lipid peroxidation terminal products, MDA content and 4‐hydroxy‐2‐nonenal (4HNE) protein levels exhibited time‐dependent augmentation in LECs following prolonged incubation (Figure 5D,E). In addition, extended incubation reduced GPX4 protein levels in a time‐dependent manner but elevated acyl‐CoA synthetase long‐chain family member 4 (ACSL4) and LPCAT3 protein levels in LECs (Figure 5F). Collectively, these results indicate that UVB irradiation results in cellular ferroptosis and upregulation of LPCAT3 in LECs.

### 3.6 Knockdown of LPCAT3 Protects LECs Against UVB Irradiation‐Induced Injury and Ferroptosis

**FIGURE 6:**
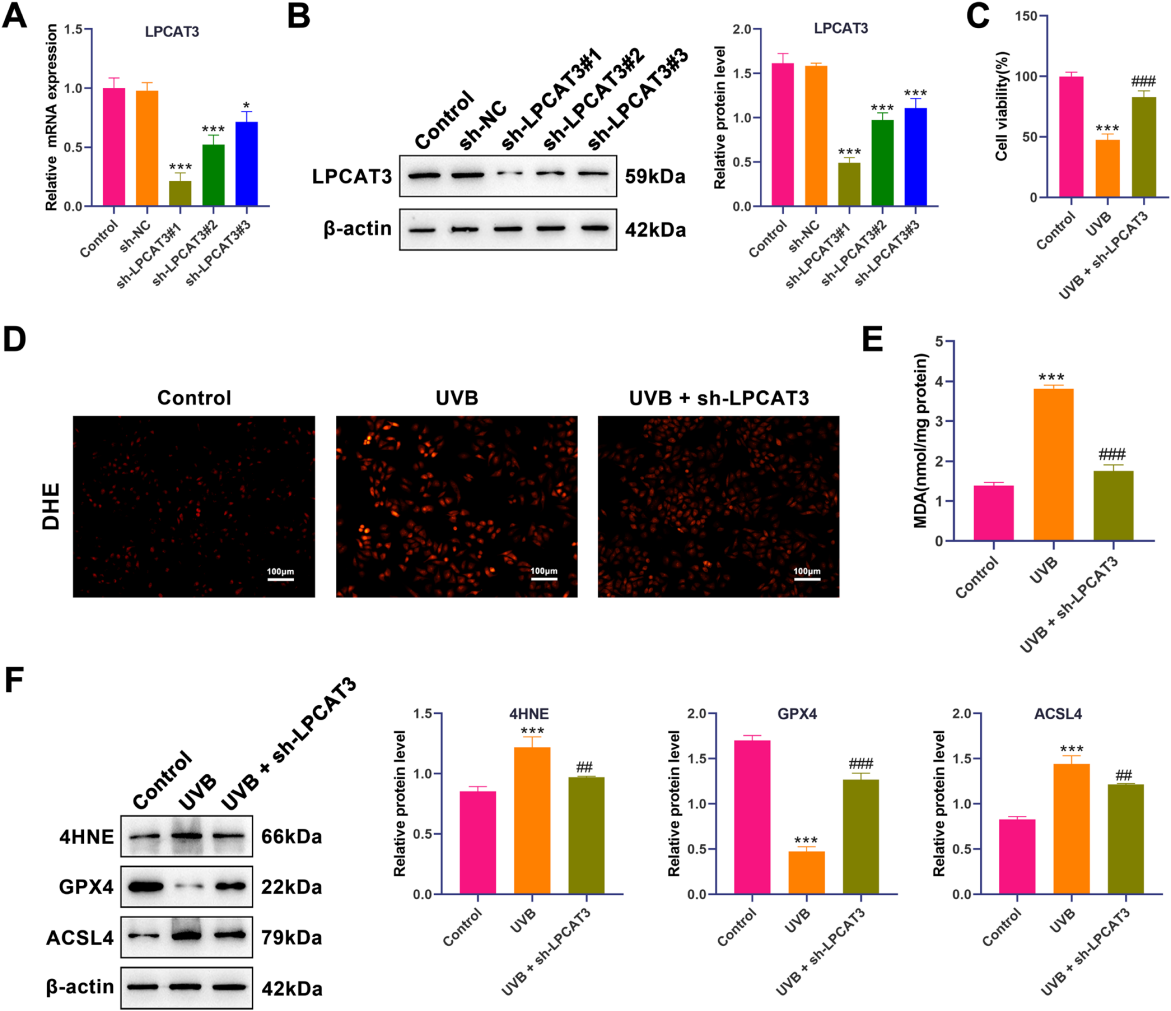
LPCAT3 deficiency attenuates UVB irradiation‐induced injury and ferroptosis in LECs. (A and B) Interference efficiencies of sh‐LPCAT3#1/sh‐LPCAT3#2/sh‐LPCAT3#3 on LPCAT3 in LECs determined by RT‐qPCR and western blot analysis. *p < 0.05 and ***p < 0.001 versus sh‐NC. (C–F) After sh‐NC or sh‐LPCAT3 transduction, LECs were irradiated with UVB (2 W/m^2^) for 48 h. (C) Cell viability detected by CCK‐8 assays. (D) ROS production analyzed by DHE staining. (E) MDA content measurements. (F) 4HNE, GPX4, and ACSL4 protein levels in LECs determined by western blot analysis. ***p < 0.001 versus control; ^##^ *p* < 0.01 and ^###^ *p* < 0.001 versus UVB. One‐way ANOVA and Tukey's test were performed to determine statistical differences (A, B, C, E, F). Data are presented as mean ± SEM.

To elucidate the function of LPCAT3 in UVB irradiation‐induced injury and ferroptosis in LECs, we knocked down LPCAT3 expression. After sh‐LPCAT3 transduction, *Lpcat3* mRNA and protein levels were significantly reduced in LECs (Figure 6A,B). sh‐LPCAT3#1, which exhibited the highest interference efficiency, was used in subsequent experiments. Functionally, the inhibitory effect of UVB irradiation on LEC viability was partially reversed by LPCAT3 knockdown (Figure 6C). Moreover, elevated ROS levels and MDA content induced by UVB irradiation were partially normalized after LPCAT3 silencing (Figure 6D,E). Additionally, LPCAT3 knockdown attenuated UVB irradiation‐mediated changes in 4HNE, GPX4, and ACSL4 protein levels in LECs (Figure 6F). Taken together, LPCAT3 knockdown shielded LECs from UVB‐triggered injury and ferroptosis.

### 3.7 LPCAT3 Silencing Alleviates UVB‐Induced Cataract Development in Rats

**FIGURE 7:**
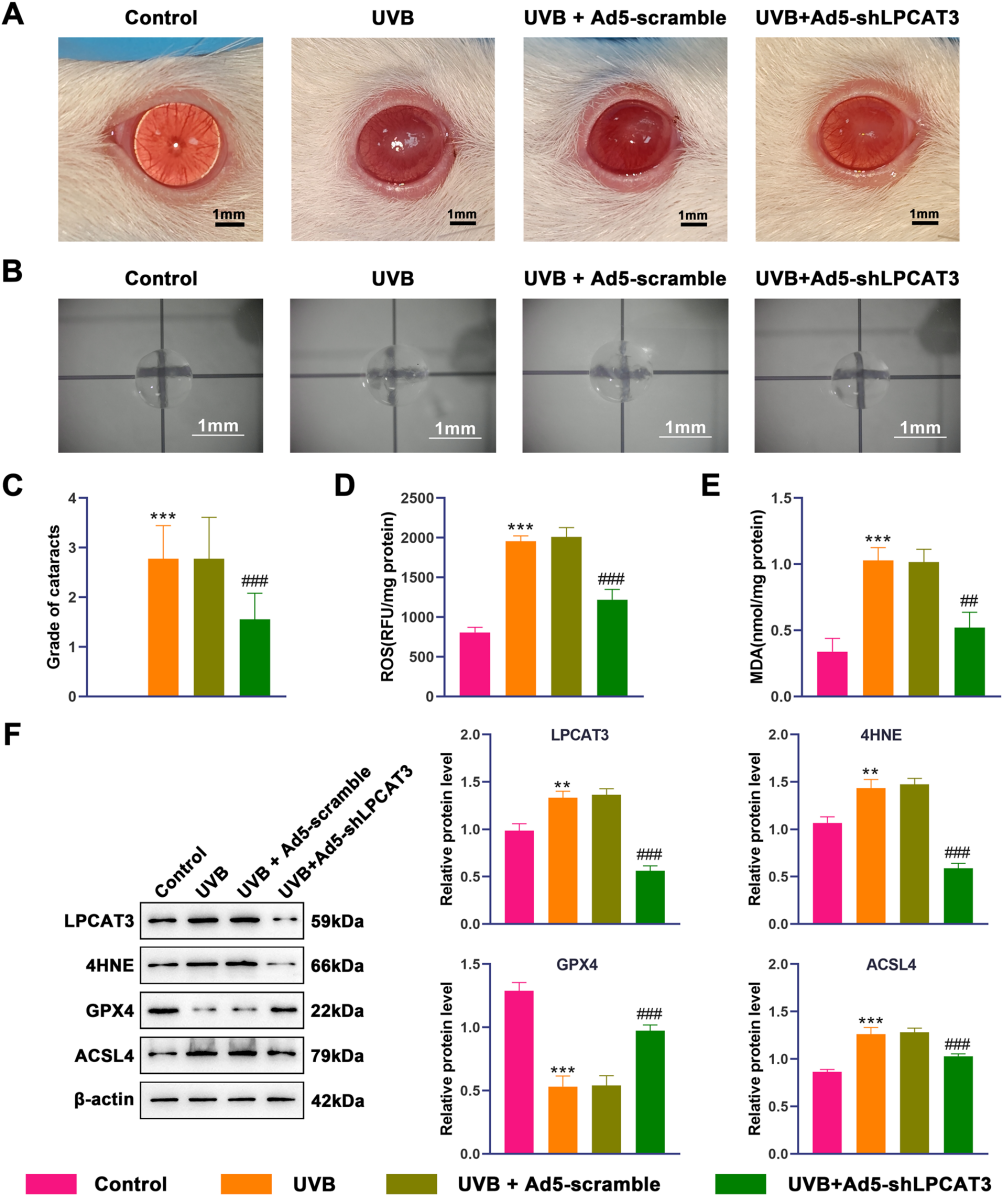
UVB‐induced cataract progression is mitigated by LPCAT3 knockdown in rats. (A) Representative images from slit‐lamp microscopy examinations of rats in each group. (B) Representative images of rat lenses taken by a stereoscope. (C) Bar graph presenting the grading of cataract scores across experimental groups of rats. (D) Quantification of lens ROS levels via fluorescence plate reader with normalization to protein content. (E) MDA concentration in rat lenses. (F) Representative western blot images and quantitative results of LPCAT3, 4HNE, ACSL4, and GPX4 protein levels in rat lens. One‐way ANOVA and Tukey's test were performed to determine statistical differences (C–F). Data are presented as means ± SEM. **p < 0.01 and ***p < 0.001 versus control; ^##^ *p* < 0.01 and ^###^ *p* < 0.001 versus UVB + Ad5‐scramble.

To verify the role of LPCAT3 in cataract formation in vivo, we established a rat model of UVB‐induced cataract by injecting Ad5‐shLPCAT3 into the vitreous cavity. Compared with control rats, UVB‐treated rats developed lens opacification with grades II–III cataracts. However, Ad5‐shLPCAT3 injection attenuated opacification in UVB‐exposed rats, reducing grades to I and II (Figure 7A–C). Significant increases in ROS production and MDA content were observed in UVB‐exposed lenses compared to controls, whereas Ad5‐shLPCAT3 administration caused a pronounced decrease in both (Figure 7D,E). As anticipated, UVB‐exposed lenses exhibited higher levels of LPCAT3, 4HNE, and ACSL4 proteins and lower protein levels of GPX4 than control lenses. However, changes in the levels of these proteins were attenuated by Ad5‐shLPCAT3 administration (Figure 7F). These findings indicated that LPCAT3 knockdown attenuated UVB‐induced cataract progression in vivo.

## 4 Discussion

This study combined transcriptomic, proteomic, and metabolomic analyses to uncover the molecular mechanisms underlying UVB‐induced cataract in rats and identified LPCAT3 as a key regulator of ferroptosis. The research results showed that LPCAT3 knockdown in in vitro and in vivo models resulted in significant reductions in UVB‐induced LEC injury and ferroptosis, as well as improved lens opacity, thus offering a novel perspective on the molecular mechanisms of UVB‐induced cataracts.

The development of UVB‐induced cataracts involves complex molecular mechanisms. This study employed a multiomics analysis of UVB‐induced lens cataracts in rats. At the transcriptomic level, 1787 DEGs revealed key pathways, including cytoskeletal remodeling, infection response, and cell cycle dysregulation. Cytoskeletal remodeling may compromise morphological and functional stability in LECs [22], whereas cell cycle dysregulation disrupts normal cellular proliferation and renewal, thereby driving cells toward senescence or apoptotic fates [23]. The upregulation of genes involved in infection responses implies UVB‐induced susceptibility to infection in the lens microenvironment, where concomitant dysregulation of local immunity could aggravate tissue injury [24], collectively representing critical pathological events in UVB‐driven cataractogenesis. Notably, several enriched pathways, including *S. aureus* infection, entailed numerous genes associated with innate immune activation and inflammatory responses, rather than representing true bacterial infection, which may explain their enrichment in this noninfectious cataract model. Proteomic analysis identified 355 DEPs, highlighting activation of the complement and coagulation cascades and aberrant DNA damage repair pathways. Activation of the complement and coagulation cascades may trigger local inflammation and tissue damage involving the lens [25, 26], which is consistent with previous studies showing that UVB irradiation induces inflammatory and oxidative stress responses in ocular tissues. Concurrently, altered expression of DNA replication‐associated proteins may reflect UVB‐induced disruption of genomic stability and the proliferative capacity of lens cells [27]. Metabolomic screening revealed 2332 significantly altered metabolites, focused on disturbances in sphingolipid and purine metabolism. Dysregulation of sphingolipid metabolism alters cell membrane composition, impacting cellular signaling [28], while disrupted purine metabolism interferes with energy supply and nucleic acid synthesis [29]. Our findings provide a comprehensive molecular landscape of UVB‐induced cataracts.

Integrated transcriptomic and proteomic analyses identified 74 codysregulated molecules enriched in pathways such as DNA replication and involved in processes such as wound healing, suggesting their potential role in forming a transcriptional–translational coregulatory network during UVB‐induced cataractogenesis. Further, cross‐omics integration with metabolomics identified four convergent pathways, including glycerophospholipid metabolism, in which LPCAT3 was significantly upregulated. LPCAT3 acts as a pivotal lipid‐metabolizing enzyme that orchestrates membrane dynamics by catalyzing phospholipid remodeling, thereby modulating membrane composition, lipid‐signaling cascades, and ferroptotic progression [30]. Emerging evidence has demonstrated the pivotal involvement of LPCAT3 in diverse pathologies. LPCAT3 promotes lipid peroxidation through the ACSL4/LPCAT3 pathway, thereby exacerbating neuronal ferroptosis [31]. Elevated LPCAT3 expression increases the susceptibility of tumor cells to ferroptosis, consequently boosting anti‐PD‐1 immunotherapy outcomes [32]. Reduced hepatic LPCAT3 expression attenuates the severity of acute liver injury caused by acetaminophen [33]. Blocking the MALT1‐LPCAT3 axis arrests cartilage degeneration and osteoarthritis progression [34]. These findings highlight the vital role of LPCAT3 in various diseases.

In this study, we investigated the role of LPCAT3 in UVB‐induced cataracts. LECs exhibited a time‐dependent reduction in viability after UVB irradiation, falling below 40% by 48 h. Although the apoptosis rate increased, no significant change occurred between 24 and 48 h, and cellular activity continued to decline. This indicates the spatiotemporal specificity of ferroptosis in cataract development; it may coexist with apoptosis in the early stages but dominates cell death during the intermediate‐to‐late phases as ROS accumulation and lipid peroxidation intensify. Following UVB irradiation, increased levels of ROS, MDA, and 4HNE, along with decreased GPX4 and elevated ACSL4 and LPCAT3 protein levels, suggested that UVB induces ferroptosis in LECs, accompanied by LPCAT3 upregulation. Silencing LPCAT3 partially reversed UVB‐induced LEC injury and ferroptosis. In animal experiments, Ad5‐shLPCAT3 injection attenuated UVB‐induced lens opacification in rats, decreased ROS and MDA levels, and modulated ferroptosis‐related protein levels. These results provide evidence for the therapeutic potential of LPCAT3 inhibition in UVB‐induced cataracts and validate its potential as a novel therapeutic target for UVB‐associated cataracts.

However, ferroptosis involves multiple regulators and signaling pathways, including classical targets (such as system xc^−^) [35] and key regulatory nodes (such as Nrf2 and p53) [36, 37]. However, whether LPCAT3 exhibits synergistic or antagonistic interactions with other ferroptosis‐related proteins remains unclear. These questions necessitate further investigation to comprehensively dissect the complex regulatory network surrounding ferroptosis and clarify the unique role and mechanistic contributions of LPCAT3. Additionally, our lens samples were pooled to generate biological replicates for multiomics analyses because of the limited tissue yield from a single lens capsule. Although this approach ensures sufficient material for multiomics analyses, it may partially obscure interindividual variability.

This study pioneered the identification of LPCAT3 as a central regulator of UVB‐induced cataracts through an integrated multiomics analysis. It drives LEC damage by triggering lipid peroxidation and ferroptosis pathways. LPCAT3 silencing effectively inhibited ferroptosis and delayed cataract progression. This work establishes a novel “lipid metabolism‐ferroptosis” theoretical framework for cataract pathogenesis and highlights LPCAT3 as a potential therapeutic target and represents a promising direction for future therapeutic strategies in cataract treatment.

## Funding

This work was supported by Study on the Protective Effect of Senescence Marker Protein 30 on the Lens of Rats Based on the Age‐Related Cataract Model of Wistar Rats (No. 8196030242).

## Conflicts of Interest

The authors declare no conflicts of interest.

## Supporting information


**Figure S1:** Workflow of the integrated multiomics analysis used in this study. Transcriptomic, proteomic, and metabolomic datasets derived from UVB‐induced cataract lenses were integrated through differential expression analysis, cross‐omics correlation, and KEGG pathway enrichment to identify key regulators associated with ferroptosis.

**Figure S2:** (A–C) Multiomics association analysis integrating metabolomic and transcriptomic datasets, highlighting cataract‐related pathways. (A) Purine metabolism. (B) Sphingolipid metabolism. (C) Nicotinate and nicotinamide metabolism.

## Data Availability

The data that support the findings of this study are available from the corresponding author upon reasonable request.
