# Supplementary figures and images for: LPCAT3 as a Potential Drug Target for Ultraviolet Radiation–Induced Cataract: Insights From Multiomics Analysis
Source: Kaohsiung J Med Sci. 2026 Apr 24:e70219. Online ahead of print. doi: 10.1002/kjm2.70219 (PMC13399675; doi:10.1002/kjm2.70219)

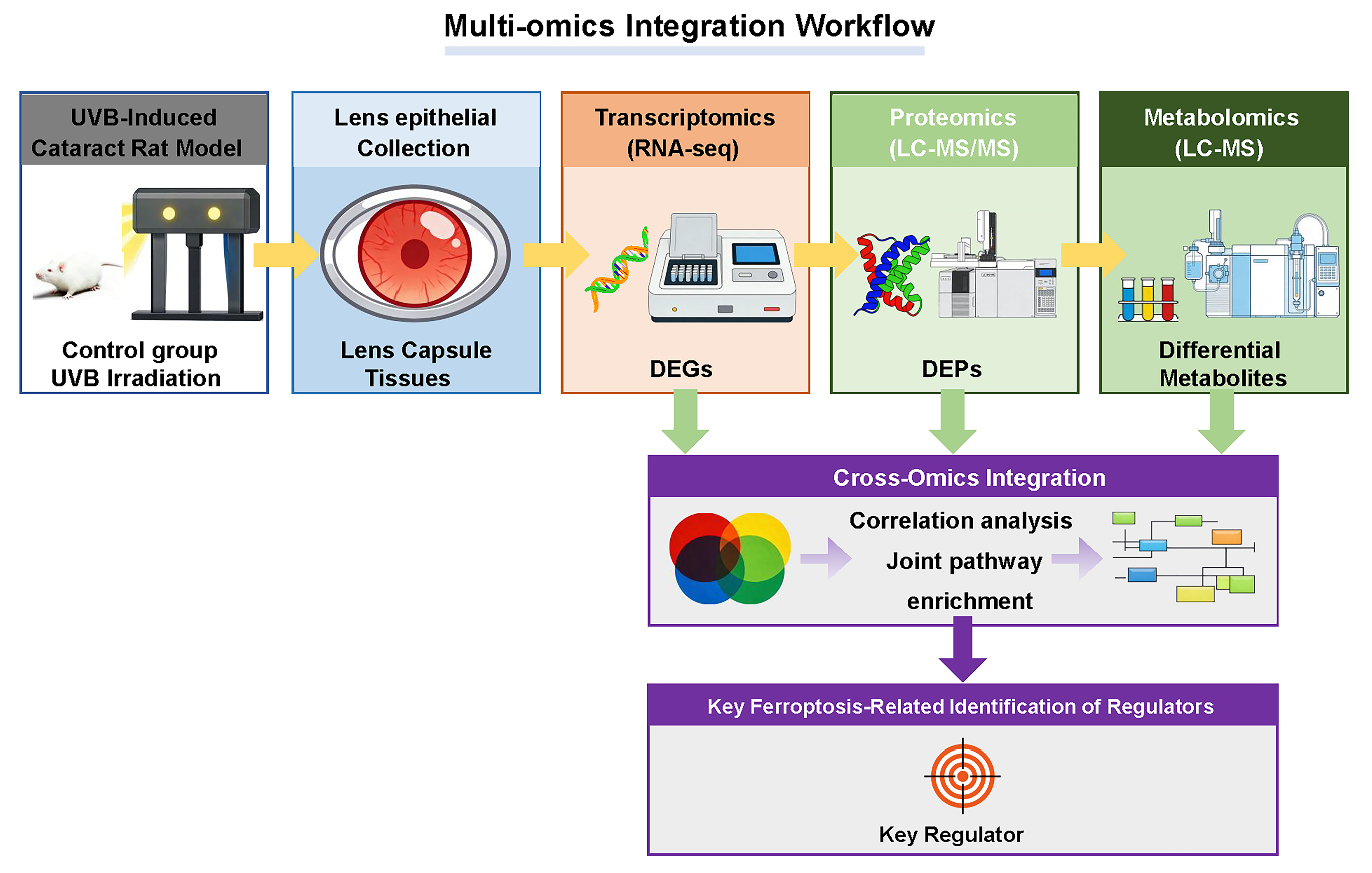

Supplement: Supplementary file 1 — Figure S1: Workflow of the integrated multiomics analysis used in this study. Transcriptomic, proteomic, and metabolomic datasets derived from UVB‐induced cataract lenses were integrated through differential expression analysis, cross‐omics correlation, and KEGG pathway enrichment to identify key regulators associated with ferroptosis. [file KJM2-9999-e70219-s001.tif]

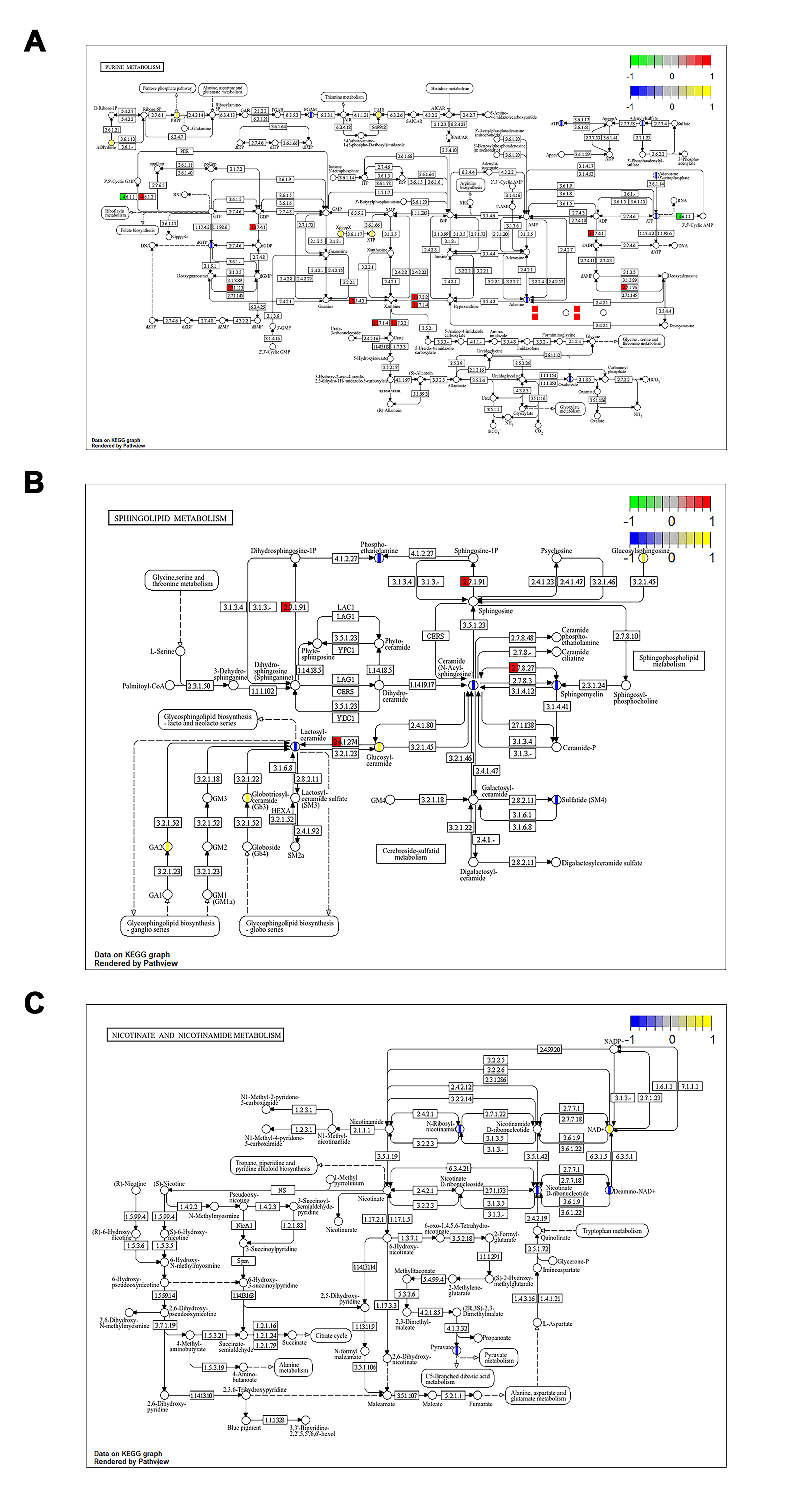

Supplement: Supplementary file 2 — Figure S2: (A–C) Multiomics association analysis integrating metabolomic and transcriptomic datasets, highlighting cataract‐related pathways. (A) Purine metabolism. (B) Sphingolipid metabolism. (C) Nicotinate and nicotinamide metabolism. [file KJM2-9999-e70219-s002.tif]
